# σ^E^-dependent small RNAs of *Salmonella* respond to membrane stress by accelerating global *omp* mRNA decay

**DOI:** 10.1111/j.1365-2958.2006.05524.x

**Published:** 2006-12

**Authors:** Kai Papenfort, Verena Pfeiffer, Franziska Mika, Sacha Lucchini, Jay C D Hinton, Jörg Vogel

**Affiliations:** 1 Max Planck Institute for Infection Biology Charitéplatz 1, 10117 Berlin, Germany; 2 Institute of Food Research, Norwich Research Park Norwich, NR4 7UA, UK

## Abstract

The bacterial envelope stress response (ESR) is triggered by the accumulation of misfolded outer membrane proteins (OMPs) upon envelope damage or excessive OMP synthesis, and is mediated by the alternative sigma factor, σ^E^. Activation of the σ^E^ pathway causes a rapid downregulation of major *omp* mRNAs, which prevents further build-up of unassembled OMPs and liberates the translocation and folding apparatus under conditions that require envelope remodelling. The factors that facilitate the rapid removal of the unusually stable *omp* mRNAs in the ESR were previously unknown. We report that in *Salmonella* the ESR relies upon two highly conserved, σ^E^-controlled small non-coding RNAs, RybB and MicA. By using a transcriptomic approach and kinetic analyses of target mRNA decay *in vivo*, RybB was identified as the factor that selectively accelerates the decay of multiple major *omp* mRNAs upon induction of the ESR, while MicA is proposed to facilitate rapid decay of the single *ompA* mRNA. In unstressed bacterial cells, the two σ^E^-dependent small RNAs function within a surveillance loop to maintain envelope homeostasis and to achieve autoregulation of σ^E^.

## Introduction

Bacteria respond to unfavourable changes in their environment by inducing specific stress regulons. Alternative sigma (σ) factors play a key role in many stress responses by redirecting RNA polymerase to the promoters of particular stress regulons. Many bacteria possess a specialized sigma factor, σ^E^, that controls aspects of pathogenesis and the development of maximal resistance to various environmental stresses (Rowley *et al*., 2006). In *Escherichia coli* and *Salmonella*, the *rpoE-*encoded σ^E^ protein is present as an inactive membrane-bound precursor in unstressed cells; upon envelope damage, controlled proteolysis cleaves the σ^E^ precursor from the membrane to yield cytoplasmic active σ^E^ (Ades *et al*., 1999; Ruiz and Silhavy, 2005). While diverse stresses (e.g. temperature shock, exposure to ethanol and antimicrobial peptides, hyperosmolarity, and entry into stationary phase) are known to induce σ^E^ (Rowley *et al*., 2006), it has been widely assumed that the actual σ^E^-activating signal is the accumulation of misfolded outer membrane proteins (OMPs) in the periplasmic space, as has been reported in exponentially growing bacteria (Mecsas *et al*., 1993; Missiakas *et al*., 1996; Raivio and Silhavy, 1999).

The σ^E^-controlled envelope stress response (ESR, also known as extracytoplasmic stress response) involves expression of > 80 transcription units of the *E. coli* and *Salmonella* genomes (Rhodius *et al*., 2006; Skovierova *et al*., 2006). While some of these genes are species-specific, most members of the σ^E^ core regulon act to synthesize and correctly assemble lipopolysaccharides and OMPs, which must be balanced to maintain envelope homeostasis.

Time-course experiments of global transcript changes upon RpoE expression identified the rapid disappearance of multiple *omp* mRNAs as a hallmark of the σ^E^ response (Rhodius *et al*., 2006). The rapid reduction of synthesis of the major OMPs represents an obvious solution to restore envelope homeostasis, as the decreased flow of OMPs to the envelope prevents further build-up of unassembled OMPs.

The factors that facilitate the rapid shut-off of OMP synthesis upon envelope stress remained unknown but we reasoned that these must involve the acceleration of *omp* mRNA decay. First, none of the hitherto identified σ^E^-controlled genes (Rhodius *et al*., 2006; Skovierova *et al*., 2006; Wade *et al*., 2006) encodes a known transcriptional repressor of *omp* genes. Second, transcriptional repression of *omp* genes would not be an effective way to mediate this rapid response, because many *omp* mRNAs are unusually stable. For example, the *ompA* message decays with a half-life of ∼ 17 min in normally growing cells (von Gabain *et al*., 1983); RpoE expression reduces its half-life to ∼ 5 min (as calculated from Fig. 1 in Rhodius *et al*., 2006). This level of stability would normally lead to the continuation of OMP synthesis for many minutes from the existing *omp* mRNAs.

**Fig. 1:**
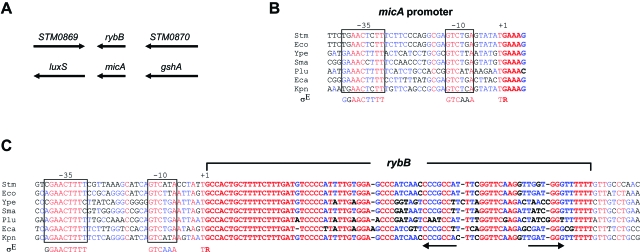
Conservation of σ^E^ consensus motifs in the promoters of *rybB* and *micA* sRNA genes. A. The *S. typhimurium micA* and *rybB* genes are located (counterclockwise) in the *luxS-gshA* and *STM0869-STM0870* (*ybjK-ybkL*) intergenic regions as previously described in *E. coli*. The black arrows indicate the orientation of the genes. B. Alignment of *micA* promoter sequences, including the first five nucleotides of the MicA-encoding sequence (in bold) of diverse γ-proteobacteria (Stm: *Salmonella typhimurium*; Eco: *Escherichia coli* K12; Ype: *Yersinia pestis*; Sma: *Serratia marcescens*; Plu: *Photorhabdus luminescens*; Eca: *Erwinia carotovora*; Kpn: *Klebsiella pneumoniae*). The −10/−35 consensus motifs of σ^E^-controlled promoters are shown below the alignment. C. Alignment of *rybB* genes, including σ^E^ motifs, of the same set of bacterial species as above. The RybB-encoding sequence is shown in bold. Arrows indicate a putative *ρ-*independent transcription terminator.

How bacterial cells actively degrade a specific set of mRNAs under stressful conditions is poorly understood. However, small non-coding RNAs (sRNAs) have recently emerged as a new class of auxiliary factors that facilitate the recognition of specific mRNAs by the general RNA decay machinery to mediate accelerated turn-over in response to adverse growth conditions. For example, iron starvation induces expression of the 90 nt RyhB sRNA, which then acts on the *trans*-encoded *sodB* mRNA to trigger its decay in a RNase E-dependent fashion (Massé and Gottesman, 2002; Massé*et al*., 2003). Similarly, the phosphosugar stress-induced 200 nt SgrS sRNA accelerates the turn-over of *ptsG* mRNA (Vanderpool and Gottesman, 2004; Morita *et al*., 2005).

About a third of the hitherto characterized enterobacterial sRNAs have been shown to target individual *omp* mRNAs (Guillier *et al*., 2006; Vogel and Papenfort, 2006), but this observation had not been linked to the σ^E^-controlled ESR. We began to make this connection because mutations of the bacterial RNA chaperone, Hfq, caused misregulation of major OMPs (Vytvytska *et al*., 1998; 2000; Ding *et al*., 2004; Sittka *et al*., 2006) and led to chronic induction of the ESR (Ding *et al*., 2004; Sittka *et al*., 2006). Hfq binds a variety of sRNAs and promotes their interaction with their target mRNAs (Valentin-Hansen *et al*., 2004), and we predicted that one or more σ^E^-regulated sRNAs would target multiple major *omp* mRNAs.

We have now identified two sRNAs, RybB and MicA, which act within the ESR of *Salmonella typhimurium*. Transcription of the RybB and MicA genes is stringently controlled by the availability of active σ^E^, and the two sRNAs are proposed to serve dual functions. First, RybB facilitates the rapid removal of an unprecedented number of major *omp* mRNAs upon induction of the σ^E^ pathway. Second, in unstressed cells, RybB and MicA form an autoregulatory loop with the σ^E^ regulon that limits OMP biogenesis to prevent the accumulation of misfolded intermediates. Collectively, the two sRNAs are likely to facilitate the remodelling of the outer membrane upon environmental challenges that require adjustments to the bacterial envelope.

## Results

### σ^E^ controls the expression of RybB and MicA sRNAs

To identify σ^E^-regulated sRNAs, we searched for σ^E^ binding motifs (Rhodius *et al*., 2006; Skovierova *et al*., 2006) in the promoter regions of the more than 50 sRNA genes predicted in the genome of *S. typhimurium* (Hershberg *et al*., 2003; Vogel and Sharma, 2005). This search yielded two candidate genes, *rybB* and *micA*. The intergenic location of these sRNA genes is conserved between *Salmonella* and *E. coli* with respect to the flanking protein-coding genes (Fig. 1A; Hershberg *et al*., 2003). The *rybB* and *micA* upstream regions each contain almost perfect matches to the −35 (GGAACTTTT) and −10 (GTCAAA) motifs of σ^E^-controlled promoters, and these elements are strongly conserved in *rybB* and *micA* genes of other γ-proteobacteria (Fig. 1B and C). In *Salmonella* the putative *rybB*−35 and −10 promoter elements each differ from the σ^E^ consensus at one position; the putative *micA* promoter has one (−35) or two (−10) mismatches with the σ^E^ consensus boxes. In both genes, the −1 and +1 positions perfectly match the σ^E^ consensus.

**Fig. 2:**
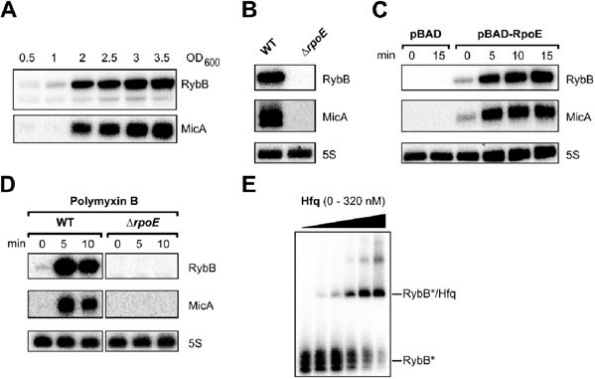
Growth phase- and σ^E^-dependent expression of *Salmonella rybB* and *micA*. A. Northern blots of total RNA probed for RybB and MicA show that the two RNAs accumulate in stationary phase cells. Samples were taken from *Salmonella* wild-type cultures throughout growth from exponential to stationary phase at the OD_600_ values indicated above the panels. RNA extraction, Northern blotting and hybridization were done as described in the *Experimental procedures* section. B–D. Northern blots show that *Salmonella rybB* and *micA* expression is strictly dependent on σ^E^. All blots were probed for 5S rRNA as loading control. B. Wild-type and isogenic *rpoE* mutant *Salmonella* were grown into stationary phase (6 h after cells had reached an OD_600_ of 2). The sRNA signals are lost in the absence of σ^E^. C. The sRNAs rapidly accumulate following RpoE expression. *Salmonella* carrying either a pBAD control plasmid (pBAD33) or pBAD-RpoE (pAC-rpoEST4) expression plasmid, in which the *rpoE* gene is cloned under an arabinose-inducible promoter, were grown to late exponential phase (OD_600_ of 1), that is when *rybB* and *micA* are not expressed. Cultures were treated with l-arabinose (0.2% final concentration) to induce RpoE expression. Aliquotes were withdrawn for total RNA preparation prior to (0 min) and at the indicated time-points (5, 10 and 15 min) following induction. The slightly elevated RybB and MicA levels at the 0 min time-point in pBAD-RpoE cells may result from leaky P_BAD_-*rpoE* transcription in the absence of l-arabinose. D. The sRNAs are rapidly induced by treatment with the antimicrobial peptide, polymyxin B, in wild-type but not Δ*rpoE* cells in late exponential phase (OD_600_ of 1.0). Total RNA was prepared prior to (0 min) and after 5 and 10 min of polymyxin B addition. E. RybB shows high affinity to Hfq protein *in vitro*. ^32^P-labelled RybB (4 nM) was incubated in the presence of 1000-fold excess of unlabelled yeast tRNA for 10 min at 37°C with increasing concentrations of purified *Salmonella* Hfq protein (from left to right: 0, 20, 40, 80, 160, 320 nM), followed by electrophoresis on a native gel. Shown is an autoradiograph of the gel.

MicA (also known as SraD) and RybB were previously identified in *E. coli* as ∼70 nt and ∼80 nt, respectively, RNAs that are detected upon entry into stationary phase (Argaman *et al*., 2001; Wassarman *et al*., 2001; Vogel *et al*., 2003). Similarly, we found that the two sRNAs are only slightly expressed in fast-growing *Salmonella enterica* serovar Typhimurium cells and strongly accumulate at stationary phase (Fig. 2A). This is consistent with a previous observation that stationary phase *Salmonella* display an elevated σ^E^ response (Testerman *et al*., 2002). In line with our prediction, RybB and MicA are not detected in a *Salmonella rpoE* mutant strain that lacks σ^E^ (Fig. 2B). However, the sRNAs rapidly accumulate following RpoE expression from the arabinose-inducible plasmid, pBAD-RpoE (Fig. 2C). In addition, envelope stress triggered by exposure to the antimicrobial peptide, polymyxin B (Humphreys *et al*., 1999), activates the *rybB* and *micA* genes in wild-type but not in *rpoE* mutant *Salmonella* (Fig. 2D).

MicA sRNA was recently shown to repress OmpA synthesis in *E. coli* in an Hfq-dependent manner (Rasmussen *et al*., 2005; Udekwu *et al*., 2005). RybB is a strongly conserved sRNA (Fig. 1C) of hitherto unknown function that was the second most abundant species in an *E. coli* sRNA library cloning screen (Vogel *et al*., 2003). *E. coli* RybB was also among the highest-scoring sRNAs in Hfq immunoprecipitation experiments (Zhang *et al*., 2003), suggesting that it acts to regulate mRNAs. We thus tested *Salmonella* RybB binding to Hfq protein *in vitro* by gel mobility shift experiments. As shown in Fig. 2E, *in vitro* synthesized RybB RNA displayed high affinity to purified *Salmonella* Hfq (Sittka *et al*., 2006), binding to the protein with an apparent *k*_D_ of 100 nM, which is similar to the level of Hfq binding of *E. coli* MicA RNA (Rasmussen *et al*., 2005). We also found that the *in vivo* stability of RybB in *Salmonella* greatly depends on Hfq. RybB decays with a half-life of ∼ 8 min in wild-type *Salmonella*, which is identical to its *E. coli* counterpart (Vogel *et al*., 2003), but its half-life is reduced to less than 1 min in *Salmonella* deleted for *hfq* (data not shown). Taken together, these results identified RybB and MicA as excellent candidates for the predicted σ^E^-regulated, Hfq-dependent sRNAs that repress major OMP synthesis upon induction of the ESR.

### RybB targets multiple OMP-encoding mRNAs

To determine the putative RybB target mRNAs, the sRNA was cloned on a plasmid under control of an arabinose-inducible P_BAD_ promoter, yielding plasmid pBAD-RybB. *Salmonella* carrying pBAD-RybB or a pBAD control vector were treated with arabinose in late exponential phase for 10 min, and the resulting global changes in transcript abundance were scored with whole-genome *S. typhimurium* microarrays. We chose this short pulse expression to only cause changes of those mRNAs with which RybB directly interacts; a similar approach has been used successfully to identify primary targets of several *E. coli* sRNAs (Massé*et al*., 2005; Tjaden *et al*., 2006).

**Fig. 3:**
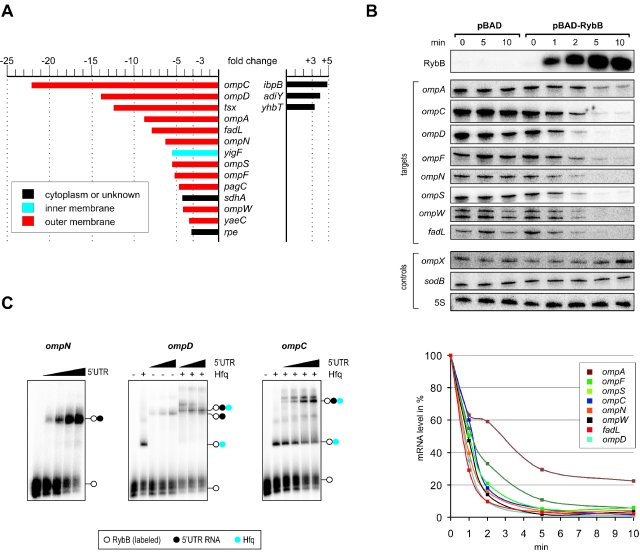
RybB targets a large set of mRNAs that encode OMPs. A. Fold changes of mRNA levels after RybB pulse overexpression. *Salmonella* carrying expression plasmid pBAD-RybB were cultured to an OD_600_ of 1.5, and RybB expression was induced with l-arabinose for 10 min. Total RNA was extracted and probed on *Salmonella* SALSA whole-genome microarrays, and data were analysed as described (Nagy *et al*., 2006). The fold changes given here are normalized to the mRNA expression changes of induced cells that carried a pBAD control vector, to correct for the effects of l-arabinose on global mRNA expression. The chart shows all mRNAs whose levels changed by > threefold, after statistical filtering (see *Supplementary material* for the entire data set). Bar colours indicate the predicted cellular localization of the encoded proteins. B. Northern blot validation of RybB-induced target mRNA decay. *Salmonella* carrying either the control pBAD vector or the pBAD-RybB expression vector were arabinose-induced at an OD of 1.5, and total RNA extracted at the time-points indicated above the panels. Northern hybridization with gene-specific probes (indicated to the left) confirmed rapid induction of RybB expression (upper panel), and a concomitant drop in the steady-state levels of eight target mRNAs (*ompA/C/D/F/N/S/W*, and *fadL*) in pBAD-RybB cells. The *ompX* and *sodB* mRNAs, whose expression did not change in the aforementioned microarray experiments, were probed as controls (panels below). Probing for 5S rRNA confirmed equal RNA loading (lower panel). A quantification of the blot signals obtained for the eight RybB target mRNAs in pBAD-RybB cells is given below. For each mRNA, the signal obtained at the 0 min time-point (prior to induction) was set to 100%. RybB expression reduces the half-life of these targets (except *ompA*) to ∼1 min. C. Gel-mobility shift assays show that RybB binds to RNA fragments derived from 5′ UTRs of three target mRNAs *in vitro*. RybB (^32^P-labelled; 5 nM final concentrations) binding assays with 5′ UTR RNAs were performed in the presence of a 1000-fold excess of yeast tRNA for 10 min at 37°C, followed by electrophoresis on a native gel, of which autoradiographs are shown. (Left panel) RybB was incubated with increasing concentrations of an unlabelled RNA fragment derived from the *ompN* 5′ UTR (from left to right: 0, 15, 30, 60, 120 nM). The positions of RybB (open circle) or the RybB/*ompN* complex (filled circle) are indicated to the right. (Middle panel) Gel mobility shifts with an unlabelled RNA derived from the *ompD* 5′ UTR (final concentration from left to right: 0, 0, 125, 250, 500, 125, 250, 500 nM). Here, Hfq was added to the individual reactions where indicated (+) at a final concentration of 30 nM; a blue circle indicates Hfq-specific mobility changes. (Right panel) Complex formation of RybB with an unlabelled RNA derived from the *ompC* 5′ UTR (final concentrations from left to right: 0, 0, 12.5, 25, 50, 100 nM). Addition of Hfq and complex formation is indicated as in the other two panels.

Of the 4716 open reading frames represented on the *Salmonella* SALSA microarray, 14 transcripts were reduced > threefold, while three mRNAs showed > threefold elevated levels (Fig. 3A and Table S2). On average, the repressed mRNAs exhibited a far higher degree of regulation, and ∼80% of these encoded OMPs. These proteins included the most abundant OMPs of *Salmonella*, i.e. OmpA, OmpC, OmpD and OmpF (Lee and Schnaitman, 1980), with the *ompC* and *ompD* mRNAs showing the strongest repression (22- and 14-fold respectively).

To confirm the transcriptomic data, we determined the kinetics of the RybB-mediated downregulation of eight *omp* target mRNAs on Northern blots (Fig. 3B). RybB expression from plasmid pBAD-RybB resulted in approximately twofold or greater reduction of these target mRNAs within 1 min, and in ≥ 10-fold reduction within 5 min (except *ompA*, which was reduced by threefold). In contrast, two RybB-independent mRNAs, *sodB* and *ompX*, that were probed as controls remained stable or increased. Likewise, arabinose induction of a strain carrying a pBAD control plasmid did not affect the abundance of the RybB targets.

Importantly, the observed downregulation is most likely to result from active mRNA degradation rather than transcriptional repression. Generally, OMP-encoding mRNAs are known to be unusually stable, e.g. the usual half-lives of the *ompC* and *ompD* mRNAs under this growth condition are 10 min and > 20 min respectively (Sittka *et al*., 2006); the action of RybB reduced these half-lives to ∼1 min (Fig. 3B). We hypothesized that RybB binds to 5′ UTR of its targets, which could block translation initiation and destabilize these mRNAs, as it was previously established for other bacterial antisense RNAs (Storz *et al*., 2004). To test this, RybB/*omp* 5′ UTR complex formation was assayed *in vitro*. Figure 3C shows that RybB readily forms complexes with the *ompN* 5′ UTR fragment, resulting in a nearly complete shift of RybB with ∼60 nM *ompN* 5′ UTR RNA. RybB complex formation was considerably weaker with the *ompC* and *ompD* 5′ UTRs, but was greatly enhanced when RybB was pre-incubated with 30 nM Hfq. While the precise RybB binding sites on its targets remain unknown, these experiments suggest that RybB promotes the decay of multiple major *omp* mRNAs by direct interaction with their 5′ UTRs, and that this regulation is Hfq-dependent.

### Limited role of other OMP-regulatory sRNAs in the ESR

**Fig. 4:**
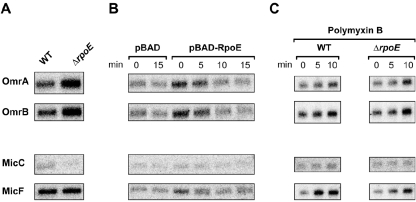
The *Salmonella omrA*, *omrB*, *micC* and *micF s*RNA genes are not members of the σ^E^ regulon. The same Northern blots as in Fig. 2B– D were probed for the four *Salmonella*sRNAs. A. Wild-type and isogenic *rpoE* mutant *Salmonella* were grown into stationary phase (6 h after cells had reached an OD_600_ of 2). B. *Salmonella* carrying either a pBAD control plasmid (pBAD33) or pBAD-RpoE (pAC-rpoEST4) expression plasmid, in which the *rpoE* gene is cloned under an arabinose-inducible promoter, were grown to late exponential phase (OD_600_ of 1). Cultures were treated with l-arabinose (0.2% final concentration) to induce RpoE expression. Aliquots were withdrawn for total RNA preparation prior to (0 min) and at the indicated time-points (5, 10 and 15 min) following induction. C. RNA samples were taken from polymyxin B-treated wild-type and Δ*rpoE* cells in late exponential phase (OD_600_ of 1). Total RNA was prepared prior to (0 min) and after 5 and 10 min of polymyxin B addition. In none of these samples, we were able to detect an RseX-specific signal.

MicA, the other σ^E^-regulated sRNA we had identified, was known to repress *ompA* mRNA in *E. coli* (Rasmussen *et al*., 2005; Udekwu *et al*., 2005). We used our transcriptomic approach to identify MicA mRNA targets in *Salmonella* and confirmed *ompA* repression, as well as showing that no other *omp* mRNA is regulated by MicA (K. Papenfort *et al*., unpubl. results). In addition to MicA, five sRNAs – MicC/F, OmrA/B and RseX – were previously shown to regulate *omp* mRNAs in *E. coli* (Mizuno *et al*., 1984; Chen *et al*., 2004; Douchin *et al*., 2006; Guillier and Gottesman, 2006). Several of these sRNAs repressed *omp* mRNAs that we found here to be targets of RybB. Specifically, both MicC and RseX were shown to act on *ompC* (Chen *et al*., 2004; Douchin *et al*., 2006), while MicF was known to target *ompF* in *E. coli* (Mizuno *et al*., 1984). By testing their *Salmonella* homologues for σ^E^-dependent expression as described above, we found that these sRNAs were not members of the σ^E^ regulon (Fig. 4). Collectively, this pointed to RybB as the major facilitator of *omp* mRNA repression in the ESR.

### RybB accelerates global *omp* mRNA decay in the ESR

**Fig. 5:**
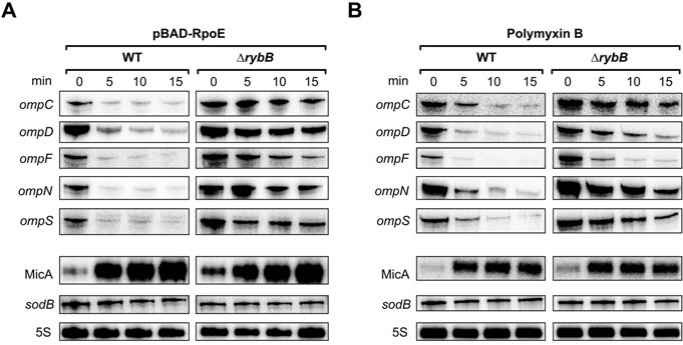
RybB facilitates *omp* mRNA decay in the ESR. A. Northern analysis of the decay of five RybB target mRNAs (*ompC*, *ompD*, *ompF*, *ompN*, *ompS*) in *Salmonella* wild-type and Δ*rybB* cells carrying plasmid pBAD-RpoE. Bacteria were grown to late exponential phase (OD_600_ of 1.5), and RpoE expression was induced with arabinose. Aliquots were withdrawn for total RNA preparation prior to (0 min) and at the indicated time-points (5, 10 and 15 min) following induction. The wild-type and Δ*rybB* samples were probed in parallel on the same blot. MicA probing shows that the ESR induction is not affected in Δ*rybB*. The blots were also probed for the ESR-independent *sodB* mRNA and 5S rRNA (loading control). B. Northern blots of *Salmonella* wild-type and Δ*rybB* cells grown to OD_600_ of 1.5 in which the ESR was induced by treatment with polymyxin B for the time indicated above the panels. Probing of the same mRNAs as in (A) shows a similar delay in the decay of the RybB-target mRNAs in *rybB* mutant *Salmonella*.

To determine the impact of the chromosomal *rybB* gene on *omp* mRNA decay in the ESR, we followed the changes of five RybB target mRNAs in wild-type and Δ*rybB* cells over time upon pBAD-RpoE expression. In wild-type *Salmonella*, RpoE expression caused a rapid decay of all these targets (Fig. 5A). Quantification of the Northern blot signals revealed a > 80% decrease within 5 min of RpoE induction, consistent with the fast disappearance of *E. coli omp* mRNAs observed by Rhodius *et al*. (2006). In contrast, target mRNA decay was delayed and incomplete in the *rybB* mutant, and exhibited some aberrant patterns, e.g. *ompC* and *ompN* mRNA levels increased within the first 5 min. The same pattern was observed when the σ^E^ pathway was activated with polymyxin B (Fig. 5B). The delayed *omp* mRNA decay in Δ*rybB* cells cannot be explained by an altered σ^E^ induction, because upregulation of the σ^E^-dependent MicA is unabated (Fig. 5). To confirm that polymyxin B activates σ^E^, which then transcribes *rybB* and *micA* to destabilize porin mRNAs, we also treated the Δ*rpoE* strain with polymyxin B and observed that downregulation of *ompD* mRNA was abrogated by the absence of σ^E^ activation (Fig. S1).

### RybB and MicA maintain envelope homeostasis

**Fig. 6:**
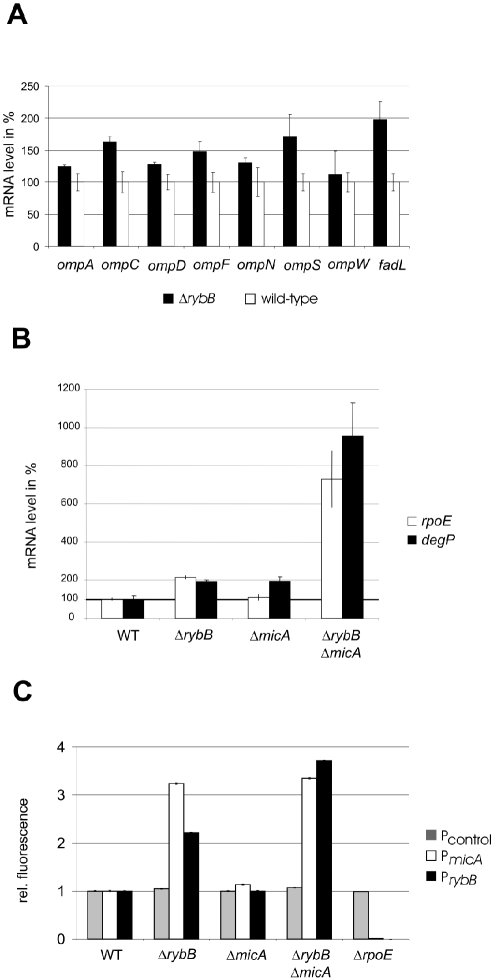
Loss of RybB and MicA functions induces the ESR. A. The Δ*rybB* mutation causes accumulation of major *omp* mRNAs in early stationary phase cultures (OD_600_ of 1.5). Shown is a comparison of major *omp* mRNA levels between wild-type and Δ*rybB* cells as determined by real-time PCR. The wild-type signals of each mRNA were set to 100%. B. Higher steady-state levels of the *rpoE* and *degP* mRNAs indicate an elevated ESR status in *rybB*, *micA* and sRNA double deletion strains. Wild-type *Salmonella* and isogenic mutant strains (as indicated) were grown to stationary phase (OD_600_ of 3), and *rpoE* and *degP* mRNA levels were determined by real-time PCR. The wild-type signals of the two mRNAs were set to 100%. C. The elevated ESR status in *rybB* and *micA* mutant cells is also apparent from a higher transcriptional activity of σ^E^-dependent promoters of these genes. Transcriptional GFP fusions to the *rybB* and the *micA* promoters were introduced in *Salmonella* wild-type and the indicated mutant strains. A GFP fusion to the constitutive P_LtetO_ promoter served as a control. Reporter activity (GFP fluorescence) was determined in cultures grown to stationary phase (OD_600_ of 3). Given are relative values, with the wild-type signals set to 1 in each case.

What are the functions of the σ^E^-dependent sRNAs, RybB and MicA, under normal growth? In the experiments described above, we observed that Δ*rybB* cells had elevated levels of several RybB target mRNAs, i.e. at the zero time point prior to σ^E^ induction (Fig. 5). This observation was confirmed by determining the levels of eight RybB target mRNAs in wild-type and *rybB* mutant *Salmonella* by quantitative real-time PCR (Fig. 6A). We predicted that absence of RybB may increase major OMP synthesis, to impact upon envelope homeostasis, and could lead to chronic ESR activation in otherwise unstressed Δ*rybB* cells. To assay the ESR status in *rybB* and/or *micA* deletion strains, we determined the levels of *rpoE* and *degP* mRNA (Fig. 6B), two sensitive markers of the σ^E^ response (Dartigalongue *et al*., 2001; Rhodius *et al*., 2006; Rowley *et al*., 2006). In the Δ*rybB* strain, approximately twofold higher levels of these σ^E^-dependent transcripts were observed. Δ*micA* cells also had elevated *rpoE* and *degP* levels, albeit not as pronounced as with Δ*rybB*. However, *rybB/micA* double deletion activated σ^E^ even further as judged by approximately sevenfold and approximately ninefold higher levels of *rpoE* and *degP* mRNAs as compared with wild-type cells. To corroborate these results, we assayed the activity of the σ^E^-dependent *rybB* and *micA* promoters in the sRNA deletion strains (Fig. 6C). To this end, the sRNA promoter regions were transcriptionally fused to a plasmid-borne *gfp* reporter gene, and the resulting plasmids were introduced into the wild-type, Δ*rybB* and/or Δ*micA*, and Δ*rpoE* strains. A reporter plasmid in which *gfp* is under control of a constitutive P_LtetO_ promoter (Lutz and Bujard, 1997) and which should not be affected by σ^E^ availability, served as control. By measuring the GFP activity of these promoter fusions, we found that the Δ*rybB* mutation – alone or in combination with Δ*micA*– resulted in several-fold activation of the *micA* and *rybB* promoters, while the *micA* deletion alone had a lesser effect. In Δ*rpoE* cells, the transcriptional activity of both the *rybB* and the *micA* promoters were reduced to near background levels, which confirmed that these sRNA genes are strictly σ^E^-dependent.

## Discussion

Small non-coding RNAs have become important players in bacterial gene regulation (Gottesman, 2004; Storz *et al*., 2005). To date, systematic genome-wide searches for these regulatory molecules have led to the identification of ∼80 sRNAs in *E. coli* (reviewed in Vogel and Sharma, 2005), the majority of which are conserved in *Salmonella* and other closely related bacterial species (Hershberg *et al*., 2003). The precise role of most of the sRNAs is still unknown, though many are only expressed under specific conditions such as stationary phase and slow growth (Argaman *et al*., 2001; Wassarman *et al*., 2001; Vogel *et al*., 2003). It has long been argued that stationary phase expression of sRNA genes may actually reflect transcriptional control by distinct stress regulons which are gradually activated upon the cessation of growth. Our new findings place two such stationary phase-specific sRNAs, RybB and MicA, at the centre of an intensely investigated stress response, based upon the alternative sigma factor, σ^E^.

σ^E^ is widespread among a diverse set of pathogenic and non-pathogenic bacteria, and becomes activated when bacterial envelope homeostasis is disturbed (reviewed in Rowley *et al*., 2006). The ESR plays fundamental roles in bacterial virulence and survival; it is induced when (i) elevated OMP production causes accumulation of misfolded OMPs in the periplasm, and (ii) the envelope requires remodelling following damage by external stresses. In both cases, the cell must avoid synthesis of the major OMPs because the ongoing translocation of these proteins cause membrane stress and so impact upon bacterial fitness. This negative link between the ESR and OMP protein production was first reported in 1993 (Mecsas *et al*., 1993), and was further highlighted by the observation of rapid removal of *omp* mRNAs upon σ^E^ induction (Rhodius *et al*., 2006), but it remained a mystery how this rapid *omp* mRNA decay could be reconciled with the extraordinarily stability of these messengers.

We examined this contradiction in *S. typhimurium*, and discovered that RybB and MicA solve this problem. We show that the σ^E^-dependent sRNA, RybB, achieves the active degradation of the bulk of *omp* mRNAs upon activation of the ESR. Consequently, a chromosomal *rybB* deletion causes the accumulation of aberrant levels of *omp* mRNAs and chronic activation of the ESR, which is accentuated by a *micA* deletion.

This is the first example of such a complex system in γ-proteobacteria, where an alternative stress Sigma factor drives the expression of regulatory sRNAs which have functionally related target mRNAs. To our knowledge, the only other case are the σ^54^-controlled, highly homologous Qrr sRNAs of *Vibrio* species, which all act on a single target mRNA that encodes a quorum-sensing regulator (Lenz *et al*., 2004). However, it is unknown whether the action of the Qrr sRNAs also regulates σ^54^ activity, as reported here for the regulation of σ^E^ activity by RybB and MicA.

The conservation of *rybB* (Fig. 1C), *micA* (Udekwu *et al*., 2005) and *rpoE* (Rowley *et al*., 2006) genes within a large group of enterobacterial species suggests that an sRNA-mediated global *omp* mRNA decay is a fundamental ESR function in many bacteria. Homology is apparent at both the transcribed and the σ^E^-dependent promoter regions, suggesting an evolutionarily conserved mechanism based upon the regulation of bacterial envelope homeostasis by RybB and MicA. A conserved regulatory pathway had been hypothesized for *E. coli* MicA when it was identified as a post-transcriptional regulator of *ompA* mRNA, but it was never suspected that a related pathway would target multiple *omp* mRNAs (Valentin-Hansen *et al*., 2004; Udekwu *et al*., 2005). Indeed, while this manuscript was in preparation, σ^E^-dependent functions of the two sRNAs have been identified in *E. coli* (Johansen *et al*., 2006; E.G. Wagner and S. Gottesman, pers. comm.). In addition, loss of Hfq which causes a dramatic activation of the ESR in *Salmonella* (Figueroa-Bossi *et al*., 2006; Sittka *et al*., 2006) was also shown to activate the *Salmonella micA* gene in a σ^E^-dependent fashion (Figueroa-Bossi *et al*., 2006). As MicA requires Hfq to repress *ompA* mRNA in *E. coli* (Rasmussen *et al*., 2005; Udekwu *et al*., 2005), and RybB is shown here both to display high affinity to Hfq and to be dependent on Hfq for binding to some of its target mRNAs *in vitro*, we speculate that the chronic envelope stress experienced by *Salmonella hfq* mutants may result from the loss of function of these two OMP-regulatory sRNAs.

**Fig. 7:**
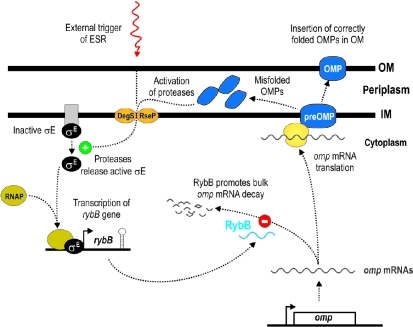
Proposed model of RybB and MicA functions in the ESR. σ^E^ activates expression of a range of phenotypes under conditions of envelope stress (Rowley *et al*., 2006). One of the key components of the ESR is a rapid decrease in mRNA levels of major OMPs (Rhodius *et al*., 2006), caused by a previously unknown mechanism. We have discovered a unique role for a sRNA, RybB, which is responsible for destabilizing many *omp* mRNA species in a σ^E^-dependent fashion. The initial triggering of the ESR is initiated by DegS which releases functional σ^E^ to activate RybB, MicA and the rest of the σ^E^ regulon. RybB is proposed to bind to many *omp* mRNAs to accelerate their decay. This halts the synthesis of bulk OMPs to protect the cell from misfolded proteins accumulating in the periplasm. We propose a similar role for the σ^E^-dependent MicA sRNA, i.e. ESR-induced *ompA* mRNA decay (not shown).

Our results support the model shown in Fig. 7. First, in normally growing cells, the two σ^E^-dependent sRNAs function within a surveillance loop to maintain envelope homeostasis. The constant flux of OMPs to the periplasm inevitably results in a fraction of misfolded OMPs, which will induce the σ^E^ pathway. Under this condition, RybB and MicA become activated and limit synthesis of OMP proteins. Of the two, RybB is the major facilitator of OMP repression, while MicA acts to repress the abundant OmpA protein. Second, if the ESR is triggered by an external stimulus, RybB and MicA function to rapidly shut off OMP synthesis. The downregulation of *omp* mRNA must be pivotal for the counteraction of envelope stress; it also occurs in the absence of RybB, albeit at a slower rate (Fig. 5), and may in part be mediated by transcriptional repression of the *omp* genes. However, it is also possible that other σ^E^-regulated sRNAs remain to be identified. We note that polymyxin B treatment mediates a faster repression of some RybB target mRNAs, e.g. *ompD* and *ompF*, than arabinose-induced σ^E^ expression from plasmid pBAD-RpoE, and that this repression is less dependent on an intact *rybB* gene. Interestingly, we found that expression of MicF, the antisense regulator of *ompF* mRNA, is induced by polymyxin B in an *rpoE*-independent manner (Fig. 4) and could contribute to downregulation of *ompF* mRNA under this condition.

These experiments clearly identified RybB as a major accelerator of bulk *omp* mRNA decay. Why do the *omp* mRNAs need to be removed so quickly? The passage of enteric pathogens such as *Salmonella* through their host continuously requires these bacteria to remodel their envelope, and the speed of stress adaptation is one secret of their success as mammalian pathogens. However, the high stability of *omp* mRNAs threatens the successful adaptation processes required for envelope remodelling. We propose that the RybB-mediated *omp* mRNA decay selectively halts OMP production to free the membrane-associated translocation and the periplasmic folding machinery for the subsequent process of cell surface remodelling. The role of such a sRNA-controlled mechanism may allow *Salmonella* to react rapidly to environmental stress encountered during infection. We note that bacteriophages commonly use OMPs as receptors for docking (Mock and Pugsley, 1982; Koebnik *et al*., 2000; Nikaido, 2003); it is possible that a rapid shut-off of receptor expression via post-transcriptional control could become a matter of survival for bacteria under phage attack.

Why did such a mechanism evolve? The answer to this question probably lies in the purpose of the σ^E^ regulon. As σ^E^ is always present in the cell, but kept inactive by the anti-sigma factor RseA, induction of this regulon is quick and flexible. We suggest that the role of a small non-coding RNA is to facilitate a similarly rapid response: unlike protease-mediated degradatory systems, sRNA-based regulation does not necessitate the time-consuming process of translation. The design of the RybB-mediated surveillance loop indicates an unexpected ‘need for speed’ in the degradation of omp mRNAs by enteric bacteria in response to environmental stress.

Furthermore, our finding that RybB alters the kinetics of *omp* mRNA removal in the ESR may have general implications for our understanding of regulatory RNAs. Although stress-related targets have been identified for numerous sRNAs (Majdalani *et al*., 2005; Storz *et al*., 2005), physiological phenotypes of sRNA deletion strains have rarely been found. This lack of phenotypes, accompanied by small effects on target abundance in the absence of the regulatory sRNA, has led to the assumption that sRNAs primarily act to fine-tune stress responses. It is clear that bacteria show an enormous robustness in the control of their most important metabolic pathways and stress responses, mediated by redundancy in regulatory, catabolic and dissimilatory pathways. Therefore, in end-point assays that measure the ability to survive a certain stress under otherwise favourable conditions, the specific contribution of a regulatory sRNA can be masked by that of a more global protein-mediated response. In this study we have used kinetic analyses of *omp* mRNA repression to pinpoint a cellular function. Future experiments will be directed at determining the consequences of RybB and MicA action for bacterial physiology and its impact on OMP biogenesis.

What is the mechanism of RybB function? Our identification of so many functionally related mRNAs as the targets of RybB is unprecedented in bacteria. The closest example of a regulatory sRNA with multiple targets is the iron starvation-induced RyhB of *E. coli*, which negatively regulates mRNAs of iron-binding proteins when iron becomes scarce (Massé and Gottesman, 2002). Structural probing of a RyhB complex with one of its targets, *sodB* mRNA, showed RyhB to base-pair to the *sodB* 5′ UTR (Geissmann and Touati, 2004), and numerous other *E. coli* sRNAs that act as repressors have been shown by structural probing and/or compensatory base-pair changes to anneal to the 5′ UTRs of their respective target mRNAs (e.g. Schmidt *et al*., 1995; Argaman and Altuvia, 2000; Chen *et al*., 2004; Vogel *et al*., 2004; Rasmussen *et al*., 2005; Udekwu *et al*., 2005; Kawamoto *et al*., 2006). In most of these cases, sRNA pairing will sequester the ribosome binding site of the target mRNA, inhibit translation, and so accelerate RNase E-mediated degradation of the target (Massé*et al*., 2003; Morita *et al*., 2005). In other words, the rapid decay of a repressed mRNA appears to be an indirect effect of translation inhibition because the half-life of bacterial mRNA is strongly affected by its association with ribosomes (Deana and Belasco, 2005). Furthermore, the SgrS and RyhB sRNAs silence translation of *pts6* and *sodB* mRNA, even in the absence of RNase E-mediated mRNA destabilization (Morita *et al*., 2006). Thus, the RybB-mediated shut-off of OMP synthesis is likely to be initiated by a block at the translational level.

Generally, *trans*-encoded antisense RNAs typically have short, imperfect sequence complementarity with their target(s) (for examples, see Wagner and Darfeuille, 2006), and are thus difficult to predict with statistical significance. The extremely rapid target decay upon pBAD-RybB expression, however, argues for direct RNA interactions. Using the TargetRNA algorithm (Tjaden *et al*., 2006) for bacterial sRNAs, Johansen *et al*. (2006) have predicted interactions of *E. coli* RybB with the 5′ UTRs of *ompC* and *ompW*, which appear to be conserved in *Salmonella*. Our preliminary results on structure probing of the RybB/*ompN* complex suggests that RybB forms an almost perfect 16 bp duplex with the 5′-coding region of *ompN* mRNA (F. Mika and J. Vogel, unpublished). However, the precise interaction sites of RybB with its many target mRNAs, as well as the contributions of Hfq and RNase E to RybB action, need to be identified on a case-to-case basis, which is the current focus of our work.

Intriguingly, the RybB/MicA functions described here bear striking similarity to the selective removal of membrane protein-encoding mRNAs within the eukaryotic unfolded protein response (UPR) (Hollien and Weissman, 2006). The UPR allows the endocytoplasmic reticulum (ER) to recover from the accumulation of misfolded proteins, when the folding capacity of the ER is exceeded. In this situation, IRE1 nuclease is activated to promote the rapid decay of a specific subset of mRNAs that are targeted to the ER (Hollien and Weissman, 2006), thus relieving the burden of misfolded proteins. By employing a sRNA (RybB) or a protein (IRE1), bacteria and eukaryotes have evolved different ways to cope with a similar problem; two distinct mechanisms that result in selective mRNA decay.

## Experimental procedures

### Oligodeoxynucleotides

Table S1 in the *Supplementary material* lists all oligodeoxynucleotides used in this study.

### Bacterial strains and plasmids

The bacterial strains and plasmids used in this study are listed in Tables 1 and 2. The *Salmonella enterica* serovar Typhimurium SL1344 strain was used as wild-type strain. Its Δ*rpoE*, Δ*rybB* and Δ*micA* derivates were constructed using the lambda-red recombinase method (Datsenko and Wanner, 2000), and primer pairs JVO-1074/JVO-1075, JVO-0279/JVO-0280 and JVO-0019/JVO-0020 respectively. All chromosomal mutations were subsequently transferred to a fresh SL1344 background strain via P22 HT105/1 int-201 transduction (Schmieger, 1971). In strain JVS-00127, the kanamycin-resistance cassette of plasmid pKD4 replaces nucleotides 1–40 of the *rybB* gene. In strain JVS-00026, the kanamycin-resistance cassette of plasmid pKD4 replaces nucleotides 1–60 of the *micA* gene. In strain JVS-01028, the chloramphenicol-resistance cassette of plasmid pKD3 replaces nucleotides 100–1133 of the *rpoE* gene. All gene deletions were verified by PCR with JVO-0021/0023 for *micA*, JVO-1076/1077 for *rpoE*, JVO-0281/0282 for *rybB*. To construct the Δ*rybB*/Δ*micA* strain JVS-01109, the kanamycin-resistance gene of JVS-00127, flanked by Flip recombinase target sites, was first removed with FLP recombinase as described in Datsenko and Wanner (2000), yielding strain JVS-01104. Subsequently, JVS-01104 served as a recipient for P22 transduction of the *micA::*Km^R^ locus from JVS-00026.

**Table 1:** Strains used in this study.

Strain	Relevant markers/genotype	Reference/source
*S. typhimurium*		
SL1344	Str^R^*hisG rpsL xyl*	Hoiseth and Stocker (1981), provided by Dirk Bumann, MPI-IB Berlin
JVS-00026	SL1344 Δ*micA::*Km^R^	This study
JVS-01028	SL1344 Δ*rpoE::*Cm^R^	This study
JVS-00127	SL1344 Δ*rybB::*Km^R^	This study
JVS-01104	SL1344 Δ*rybB*	This study
JVS-01109	SL1344 Δ*rybB*Δ*micA::*Km^R^	This study
*E. coli*		
TOP10F′	F′{*lac*I^q^ Tn*10* (Tet^R^)} *mcr*A Δ(*mrr-hsd*RMS*-mcr*BC) Φ8*0lac*ZΔM15 Δ*lac*X74 *deo*R *rec*A1 *ara*D139 Δ(*ara-leu*)7697 *gal*U *gal*K *rps*L *end*A1 *nup*G	Invitrogen

**Table 2:** Plasmids used in this study.

Name	Relevant fragment	Comment	Origin/marker	Reference
pJV861-9	P_LtetO_-*lacZ*::*gfp*	*lacZ* transcriptional GFP fusion plasmid	pSC101*/Cm^R^	Urban and Vogel (2006)
pJV783-1	P_*micA*_*-gfp*	*micA* transcriptional GFP fusion plasmid	pSC101*/Cm^R^	This study
pJV784-25	P_*rybB*_*-gfp*	*rybB* transcriptional GFP fusion plasmid	pSC101*/Cm^R^	This study
pJV990	*luc*	pZE12-luc derivate, ColE1 origin exchanged pSC101* of pZS*24-MCS-1	pSC101*/Amp^R^	This study and Lutz and Bujard (1997)
pAS0046	*gfp*	Transcriptional fusion vector, based on pJV859-8	pSC101*/Cm^R^	Sittka *et al*. (2006)
pJV790	P_LtetO_-*lacZ*::*gfp*	*lacZ* transcriptional GFP fusion plasmid	pSC101*/Amp^R^	This study
pJV791	P_*micA*_*-gfp*	*micA* transcriptional GFP fusion plasmid	pSC101*/Amp^R^	This study
pJV792	P_*rybB*_*-gfp*	*rybB* transcriptional GFP fusion plasmid	pSC101*/Amp^R^	This study
pBAD/Myc-His A		pBAD expression plasmid	pBR322/Amp^R^	Invitrogen
pKP8-35 (pBAD)		pBAD control plasmid, expresses ∼50 nt nonsense RNA derived from *rrnB* terminator	pBR322/Amp^R^	This study
pKP17-1 (pBAD-RybB)	*rybB*	RybB expression plasmid, *rybB* is controlled by the plasmid-borne P_BAD_ promoter	pBR322/Amp^R^	This study
pBAD33		pBAD expression plasmid	pBR322/Cm^R^	Guzman *et al*. (1995)
pAC-rpoEST4 (pBAD-RpoE)	*rpoE*	RpoE expression plasmid, *rpoE* is controlled by the plasmid-borne P_BAD_ promoter	pBR322/Cm^R^	Miticka *et al*. (2003)
pKD3		Template for mutant construction; carries chloramphenicol-resistance cassette	oriRγ/Amp^R^	Datsenko and Wanner (2000)
pKD4		Template for mutant construction; carries kanamycin-resistance cassette	oriRγ/Amp^R^	Datsenko and Wanner (2000)
pKD46	P_araB_-*γ-β-exo*	Temperature-sensitive lambda-red recombinase expression plasmid	oriR101/Amp^R^	Datsenko and Wanner (2000)
pCP20		Temperature-sensitive FLP recombinase expression plasmid	oriR101/Amp^R^, Cm^R^	Datsenko and Wanner (2000)

### Plasmid construction

Plasmids for l-arabinose-inducible expression of *rybB* and *micA* genes were constructed by amplification of plasmid pBAD-His-myc [cycling parameters: 95°C/30′, 25× (95°C/10′, 59°C/30′, 72°C/2′), 72°C/10′] with primers JVO-0900/0901 (JVO-0901 introduces an XbaI restriction site upstream of the *rrnB* terminator sequence). The PCR product was digested with XbaI and DpnI. For amplification of the *rybB* insert, the sense primer (JVO-0906) starts with the sRNA +1 site (as previously mapped in *E. coli*; Argaman *et al*., 2001; Vogel *et al*., 2003) and carries a 5′ phosphate modification. The antisense primer (JVO-0282) binds close to the 3′ end of the *rybB* terminator, and adds an XbaI site to its sequence. Following amplification and PCR product digestion with XbaI, the vector- and sRNA-derived PCR products were ligated with T4 DNA ligase (5′ blunt end/3′ XbaI site), yielding plasmids pKP17-1 (pBAD-RybB). Correct inserts were confirmed by sequencing of the plasmids with vector primers, pBad-FW and pBad-REV.

Amplification of plasmid pBAD-His-myc using primers JVO-0900/JVO-0901, PCR product digestion with DpnI, and religation with T4 DNA ligase yielded the pBAD control plasmid, pKP8-35. P_BAD_ expression in pKP8-35 results in a ∼50 nt nonsense transcript derived from the *rrnB* terminator sequence.

The transcriptional P_*micA*_*-gfp* fusion plasmid (pJV783-1) was constructed by digestion of pAS0046 (Sittka *et al*., 2006) with AatII/NheI and ligation with a PCR product amplified with JVO-1230/-1231. To generate the P_*rybB*_*-gfp* transcriptional fusion plasmid (pJV784-25), pAS0046 was digested as described above and ligated with a PCR fragment amplified with JVO-1232/-1233. To replace the *cat* (chloramphenicol)-resistance cassette in both plasmids, an AatII/AvrII-generated fragment was replaced by the *amp* (ampicillin)-resistance cassette from pJV990, yielding plasmid pJV791 (transcriptional P_*micA*_*-gfp* fusion) and pJV792 (transcriptional P_*rybB*_*-gfp* fusion). For construction of the control plasmid, the *cat* gene was replaced in the same way in plasmid pJV861-9 (encoding a short *E. coli lacZ* fragment fused to *gfp* under a P_LtetO_ promoter; Urban and Vogel, 2006) resulting in pJV790. Competent *E. coli* TOP10 F′ cells (Invitrogen) were used for all cloning procedures.

### Bacterial growth, l -arabinose induction and polymyxin B treatment

Growth in Luria–Bertani (LB) broth (220 rpm, 37°C) or on LB plates at 37°C was used throughout this study. SOC medium was used to recover transformants after heat-shock or electroporation and prior to plating. Antibiotics (where appropriate) were used at the following concentrations: 100 μg ml^−1^ ampicillin, 50 μg ml^−1^ kanamycin, 20 μg ml^−1^ chloramphenicol. For RybB, MicA and RpoE expression from pBAD-derived plasmids, cultures were treated with l-arabinose (final concentration of 0.2%). Polymyxin B (Sigma-Aldrich; #P9602–1VL) was used at a final concentration of 1 μg ml^−1^ (Fig. 2D) or 5 μg ml^−1^ (Fig. 5B).

### Microarray experiments

Strain SL1344 was transformed with plasmids pKP8-35 (control) and pKP17-1 (pBAD-RybB), and grown in liquid culture from single colonies to an OD_600_ of 1.5, at which sRNA expression was induced with l-arabinose for 10 min. RNA extraction and data generation are described in the *Supplementary material*.

### Northern blot analysis

Overnight cultures were diluted 1/100 in fresh medium and grown to the indicated cell densities (OD_600_). Culture aliquots were removed and mixed with 0.2 vol. of stop solution (5% water-saturated phenol, 95% ethanol), and snap-frozen in liquid nitrogen. After thawing on ice, bacteria were pelleted by centrifugation (2 min, 16 000 rcf, 4°C), and RNA was isolated using the Promega SV total RNA purification kit as described at http://www.ifr.ac.uk/safety/microarrays/protocols.html(Kelly *et al*., 2004) or using the Trizol method (in the case of the RpoE overexpression and polymyxin B experiments shown in Figs 2 and 4). The purified RNA was quantified on a Nanodrop machine (NanoDrop Technologies).

RNA samples (∼5 μg) were denatured for 5 min at 95°C in RNA loading buffer (95% [v/v] formamide, 0.1% [w/v] xylene cyanole, 0.1% [w/v] bromphenol blue, 10 mM EDTA), separated on 8.3 M urea/5% polyacrylamide gels, and transferred to Hybond-XL membranes (GE Healthcare) by electroblotting (1 h, 50 V, 4°C) in a tank blotter (Peqlab, Germany). Following pre-hybridization of the membranes in Rapid-hyb Buffer (GE Healthcare), [^32^P]-labelled gene-specific probes (Table S3) were added and hybridization was performed at the temperatures given in Table S3. After hybridization for 2 h, membranes were rinsed at room temperature in 2× SSC/0.1% SDS, followed by washing in three subsequent 15 min steps in SSC (2×, 1× or 0.5× respectively)/0.1% SDS solutions (at the hybridization temperature). Membranes hybridized with end-labelled oligodeoxyribonucleotides were rinsed in 5× SSC followed by three wash steps at 42°C in SSC (5×, 1× and 0.5× respectively). Signals were visualized on a phosphorimager (FLA-3000 Series, Fuji), and band intensities quantified with AIDA software (Raytest, Germany).

### Hybridization probe generation

Primers for template amplification are listed in Table S3. Standard polymerase chain reactions were carried out on genomic DNA. Double-stranded DNA probes (PCR products) were random-labelled in the presence of [^32^P]-α-dCTP using the Rediprime II labelling kit (GE Healthcare). Single-stranded RNA probes (riboprobes) were generated from PCR fragments (a T7 RNA polymerase promoter sequence was added by the antisense primer) in the presence of an excess of [^32^P]-α-UTP over unlabelled UTP using the Ambion T7 polymerase Maxiscript kit. DNA oligonucleotides were labelled with [^32^P]-γ-ATP using T4 polynucleotide kinase (Fermentas). All labelled probes were purified over G50 columns (GE Healthcare) to remove unincorporated nucleotides prior to hybridization.

### Synthesis, purification and labelling of RNA for *in vitro* binding assays

DNA templates carrying a T7 promoter sequence were generated by PCR using genomic DNA and primers as listed in Table S1. For RybB oligonucleotides JVO-1242 (adds T7 promoter)/-1243 (binds in the terminator region) were used. For the 5′ UTRs of *ompN* primer pair JVO-1244/-1245 [the fragment covers the *ompN* region from positions −73 to +89 (30th aa) relative to the start codon], *ompC* primer pair JVO-1246/JVO-1247 [the fragment covers the *ompC* region from positions −78 to +100 (33rd aa) relative to the start codon], and *ompD* primer pair JVO-1186/JVO-1058 [the fragment covers the *ompD* region from positions −69 to +118 (39th aa) relative to the start codon]. Primers JVO-1244, JVO-1246 and JVO-1186 add a T7 promoter sequence to the 5′ ends of the respective PCR fragments.

*In vitro* transcription was performed using the MEGAscript High Yield Transcription Kit (Ambion, #1333), followed by DNase I digestion (1 unit, 15 min, 37°C). Following extraction with phenol : chloroform : isopropanol (25:24:1 v/v), the RNA was precipitated overnight at −20°C with 1 vol. of isopropanol. RNA integrity was checked on a denaturing polyacrylamide gel.

To label *in vitro* synthesized RybB RNA, 20 pmol RNA was dephosphorylated with 10 units of calf intestine alkaline phosphatase (New England Biolabs) in a 20 μl reaction at 37°C for 1 h. Following phenol extraction, the RNA was precipitated overnight with ethanol/sodium acetate and 20 μg glycogen. The dephosphorylated RNA was 5′-end-labelled with ^32^P-γATP (20 μCi) and 1 unit of polynucleotide kinase (New England Biolabs) for 30 min at 37°C in a 20 μl reaction. Unincorporated nucleotides were removed using Microspin G-50 Colums (GE Healthcare), followed by purification of the labelled RNA on a denaturing polyacrylamide gel (6%/7 M urea). Upon visualization of the labelled RNA by exposure on a phosphorimager, the RNA was cut from the gel and eluted with RNA elution buffer (0.1 M sodium acetate, 0.1% SDS, 10 mM EDTA) at 4°C overnight, followed by phenol extraction and precipitation as before.

### Electrophoretic mobility shift assays

RybB/Hfq binding assays were performed in 1× structure buffer [100 mM Tris pH 7, 1 M KCl, 100 mM MgCl_2_, provided along with RNase T1 (#2283) from Ambion, USA] as follows. 5′-labelled RybB RNA (∼4 nM final concentration in binding reaction) and 1 μg of yeast RNA (final concentration: 4.3 μM) were incubated in the presence of Hfq (concentrations as given in the figure legend) in 10 μl reactions at 37°C for 10 min. The Hfq dilutions, calculated for the Hfq hexamer were prepared in 1× dilution buffer (1× structure buffer with 1% glycerol, 0.1% Triton X-100). *Salmonella* Hfq protein was prepared as outlined in Sittka *et al*. (2006).

RybB/5′ UTR binding assays were performed as above in the absence of Hfq (*ompN*) or the presence of 30 nM Hfq (*ompC*, *ompD*). The final concentrations of 5′ UTR RNAs are given in legend to Fig. 3G. Prior to gel loading, the binding reactions were mixed with 3 μl of loading buffer (50% glycerol, 0.5× TBE, 0.2% bromphenol blue), and electrophoresed on native 6% polyacrylamide gels in 0.5× TBE buffer at 250 V at 4°C for 3 h. Gels were dried, and analysed using a phosphorimager (see above).

### Quantitative RT-PCR

To determine the mRNA levels of wild-type and Δ*rybB* (JVS-0127) cells overnight cultures were diluted 1:100 and cells were grown to an OD_600_ of 3 [*rpoE* and *degP (htrA*)] or 1.5 (*ompA*, *ompF*, *ompC*, *ompD*, *fadL*, *ompS*, *ompW*, *ompN*). Two millilitre aliquots were removed and treated with 0.2 vol. of stop solution (95% EtOH; 5% water-saturated phenol). Cells were snap-frozen in liquid nitrogen and stored at −80°C until RNA extraction. RNA extraction was carried out as described above (Promega SV total RNA purification kit) and RNA concentrations were determined on a Nanodrop machine (NanoDrop Technologies). The relative amount of target mRNA was determined by quantitative real-time PCR using Quantitect™ SYBR® Green RT-PCR Kit following manufacturer's instructions (Qiagen) and calculating standard deviations as outlined in Paland *et al*. (2006). Specific primer pairs for *rpoE* (JVO-1236/1237), *degP* (JVO-1234/1235), *ompA* (JVO-1090/1091), *ompF* (JVO-1328/1329), *ompC* (JVO-1092/1093), *ompD* (JVO-1094/1095), *fadL* (JVO-1393/1394), *ompS* (JVO-1332/1333), *ompW* (JVO-1334/1335) and *ompN* (JVO-1330/1331) were designed using the *PRIMER EXPRESS*™ software (Applied Biosystems). *rfaH* (JVO-1117/1118) was used as an internal standard.

### Fluorescence measurements

Strains carrying the GFP fusion plasmids were inoculated 1:100 in LB medium. At the indicated cell density, 3 × 100 μl of culture was transferred to a 96 well plate, and fluorescence was measured at 37°C using a VICTOR^3^™ machine (1420 Multilabel Counter, Perkin Elmer). All experiments were done in triplicates. Plasmid pJV859-8, which expresses GFP from a constitutive P_LtetO_ promoter, served as a control. Strains without a plasmid served as background control (autofluorescence). The detailed protocol of fluorescence measurement is described in Urban and Vogel (2006).

## Supplementary material

The following supplementary material is available for this article online:

Fig. S1 An rpoE deletion abrogates the rapid downregulation of ompD mRNA upon polymyxin B treatment.

Table S1 Oligodeoxynucleotides used in this study

Table S2 Microarray results of pBAD-RybB expression.

Table S3 Probes for Northern detection and hybridization conditions.

This material is available as part of the online article from http://www.blackwell-synergy.com
