# Supplementary material for: σE-dependent small RNAs of Salmonella respond to membrane stress by accelerating global omp mRNA decay
Source: Mol Microbiol. 2006 Dec;62(6):1674–88. doi: 10.1111/j.1365-2958.2006.05524.x (PMC1804206; doi:10.1111/j.1365-2958.2006.05524.x)

Supporting Online Material

σ^E-dependent sRNAs of *Salmonella* respond to membrane stress by accelerating global *omp* mRNA decay

Kai Papenfort, Verena Pfeiffer, Franziska Mika, Sacha Lucchini, Jay C.D. Hinton, Jörg Vogel[#]

[#] To whom correspondence should be addressed. E-mail: vogel@mpiib-berlin.mpg.de

This supplement contains:

Supplementary Materials and Methods

Fig. S1

Tables S1, S2, S3

Supplementary Materials and Methods

Sample preparation for microarray experiments

3 OD aliquotes were removed, treated with 0.2 vol of stop solution (95% EtOH; 5% water saturated Phenol), snap-frozen in liquid nitrogen and stored at -80°C. For RNA extraction the cells were thawed on ice and pelleted by centrifugation (10 min, 4000 rpm, 4°C). RNA was isolated using the Promega SV total RNA purification kit as described at www.ifr.ac.uk/safety/microarrays/protocols.html. RNA concentration was determined on a Nanodrop machine (NanoDrop Technologies), and RNA quality was assessed using the RNA Laboratory-on-a-Chip (Agilent Technologies, Palo Alto, CA) as directed by the manufacturers. Experiments were performed in triplicates.

Microarray data generation

The microarrays used in this study include PCR products of all the genes present in the sequenced *S. typhimurium* strain LT2. In addition, we added 229 genes specific to *S. typhimurium* strain SL1344. Details of all the amplicons can be found at http://www.ifr.ac.uk/Safety/MolMicro/pubs.html. Our experimental design involves the use of *Salmonella enterica* serovar Typhimurium genomic DNA as the co-hybridized control for one channel on all microarrays. This method has the advantage of allowing the direct comparison of multiple samples. Total RNA and chromosomal DNA were labelled by random priming according to the protocols described at http://www.ifr.bbsrc.ac.uk/safety/microarrays/protocols.html. Briefly, 16 µg RNA were reverse transcribed and labelled with Cy3-conjugated dCTP (Pharmacia) using 200U of Stratascript (Stratagene) and random octamers (Invitrogen). Chromosomal DNA (400 ng) was labelled with Cy5-dCTP using the Klenow fragment. After labelling, each Cy3-labelled cDNA sample was combined with Cy5-labelled chromosomal DNA and hybridised to a microarray overnight at 65ºC. After hybridisation, slides were washed and scanned using a GenePix 4000A scanner (Axon Instruments, Inc.). Fluorescent spots and the local background intensities were identified and quantified using Bluefuse software (BlueGnome, Oxford). To compensate for unequal dye incorporation, data centring to zero was performed for each block (one block being defined as the group of spots printed by the same pin). We considered genes to be differentially expressed if they displayed ≥ 3-fold changes in all replicates and were statistically significantly different using Significance Analysis of Microarrays (Tusher et al. 2001). The final list included a number of genes whose gene expression is highly sensitive to small changes in growth conditions (data not shown). The consequence is that those genes have a higher probability to be false positives. Quantitative RT-PCR on independent RNA samples confirmed that the gene expression level of those genes (*cydA, napC, narK, nirB*) was not dependent on RybB (data not shown). Those genes were therefore excluded from further analysis. Data visualisation and data mining was performed using GeneSpring 7.3 (Agilent).

Supplementary Tables & Figures

Table S1: Oligodeoxynucleotides used in this study.

Name	Sequence in 5'→ 3' direction
JVO-0019	AAATAAACTGAACTCTTTGTTCCGGGGCGAGTCTGAGTATATGAAAGACGTGTAGGCTGGAGCTGCTTC
JVO-0020	GGCGGATACCGAGCCGTTTGCCGCGTGGCTTGCAAAACACGCCTGACCCAGGTCCATATGAATATCCTCCTTAG
JVO-0021	GTTTTTCTCGAGCAGATCAAACACGGTGATT
JVO-0023	GTTTTTTCTAGAGCCGCTGGAGATTTTACA
JVO-0236	GAAAGACGCGCATTTGT
JVO-0266	GTTTTTTTTTAATACGACTCACTATAGGGAGGCACGGAGTGGCCAAA
JVO-0279	GTTTGCCTTTAAGTGAAAAAATTTTGCCAATAGGTCGAACTTTTCGTTAAGGTCCATATGAATATCCTCCTTAG
JVO-0280	CGTTGGGCAACAAAAAACCCACCAACCTTGAACCGAAATGGCGGGGTTGAGTGTAGGCTGGAGCTGCTTC
JVO-0281	GTTTTTCTCGAGCGGCGATGGAAATCAT
JVO-0282	GTTTTTTCTAGACGCCGGAGTGTCAAT
JVO-0322	CTACGGCGTTTCACTTCTGAGTTC
JVO-0397	CGGTAGAGTAACTATTGAGCAGAT
JVO-0398	GTTTTTTTTTTAATACGACTCACTATAGGGAGGCCTAACCAGTCGTAGC
JVO-0430	GTTTTTATGCATAGACACATAAAGACACCAAACTC
JVO-0717	GTTTTTGCTAGCTGGTACCAGGAGGG
JVO-0719	GTTTTATGCATGCCGACTGGTTAATGAG
JVO-0900	GGAGAAACAGTAGAGAGTTGC
JVO-0901	TTTTTTCTAGATTAAATCAGAACGCAGA
JVO-0906	5'P-GCCACTGCTTTTCTTTGA
JVO-0932	CCACCGCTCAATTTGC
JVO-0933	CGATCTCTTCCAGCGATT
JVO-1057	GTTTTTTTTAATACGACTCACTATAGGCCATTGACAAACGC
JVO-1058	CGTGAACTTTACCGTACA
JVO-1074	AGATCAGAAAGCCTTTAACTTACTGGTAGTGCGCTACCAGCATAAAGTGGTCCATATGAATATCCTCCTTAG
JVO-1075	TATCTCTTCATAGCTCAGGCCATCCAGCTCCCGTAAGGTGATTGCCATACGTGTAGGCTGGAGCTGCTTC
JVO-1076	GATAAGACCTGTCTACAACATGA
JVO-1077	TAACTCTCCCAGGTTTTCTG
JVO-1090	CTTCATTCACAATGATGGCCC
JVO-1091	TCGTAGCCCATTTCAAAGCC
JVO-1092	CCTACGGCGCTGACAACTTTA
JVO-1093	TAACGCGAAGTCCAGACCATC
JVO-1094	ACGTTCTGCCAGAGTTTGGTG
JVO-1095	CCAGGCCAAAGAAGTCAGTGTT
JVO-1117	TCAGCCATTTTGTGCGCTT
JVO-1118	TTCAGGATCGACAACGCCTT
JVO-1186	TTTTCTCGAGTTAATACGACTCACTATAGGCCATTGACAAACG
JVO-1187	GACACCGTGAATCGCA
JVO-1188	GTTTTTTTTTTAATACGACTCACTATAGGGAGGCAGTGATGCCGTAGT
JVO-1195	GGATGCCTTTGATTCAAC
JVO-1196	GTTTTTTTAATACGACTCACTATAGGGAGGTCTGATCGCCATCTT
JVO-1197	GAACAAATGTGATCTGTATTAGATC
JVO-1198	GTTTTTTTAATACGACTCACTATAGGGAGGTCTGACGGTTGCC
JVO-1199	TCCATGAACTTCATAGAATAGTATC
JVO-1200	GTTTTTTTAATACGACTCACTATAGGGAGGTCGAGTTTGTTGCC
JVO-1202	GTTTTTTTAATACGACTCACTATAGGGAGGCTTTACCGTACAGATCCAG
JVO-1203	GCCACTGGTCTGATTTCTA
JVO-1204	GTTTGTTTAATACGACTCACTATAGGGAGGCCAGAGGAAGAAAAT
JVO-1205	GTTGATGGGCTCCACAA
JVO-1230	GTTTTGACGTCGCAGATCAAACACGGT
JVO-1231	GTTTTGCTAGCGTCTTTCATATACTCAGAC
JVO-1232	GTTTTGACGTCGCGCAGTAATATTCCA
JVO-1233	GTTTTGCTAGCAGTGGCAATAGGTATG
JVO-1234	AGGTTTGGCATTGTCGCCT
JVO-1235	CTTTTTCGAGCATCGGTGC
JVO-1236	ACTATTGAGTCCCTCCCGGAAG
JVO-1237	ACCGGACAATCCATGATAGCC
JVO-1242	GTTTTTTTTAATACGACTCACTATAGGCCACTGCTTTTCTTTGA
JVO-1243	AACCCACCAACCTTGAA
JVO-1244	GTTTTTTTTAATACGACTCACTATAGGATGCCTTTGATTCAA
JVO-1245	GTTGCCGTCTTTGTTATAAAT

JVO-1246	GTTTTTTTTAATACGACTCACTATAGGCCGACTGGTTAATGAG
JVO-1247	GGTCTAATTTGTTGCCGT
JVO-1328	CGCAAACGCAGCAGAAATT
JVO-1329	TTTTACTATCGCCGGTCGTTG
JVO-1330	GTCTGAACTTCGCGTTGCAAT
JVO-1331	CACCGTTAGCGTTCTTCACGT
JVO-1332	CCTGTACGGCAAAGTTGATGG
JVO-1333	CGTTAATCTGCGTTTCGCCT
JVO-1334	ATTCCAGCAGCAAAGTGCGT
JVO-1335	GGACAGCCCGGCATTTTTA
JVO-1393	CGAACGTCCATTTTGTCGC
JVO-1394	CCGGCGTATGTGTCGTTAAAC
PBADFW	ATGCCATAGCATTTTTATCC
PBADREV	TTATCAGACCGCTTCTGC

Table S2: Microarray results (provided as an separate EXCEL file).

Table S3: Probes for Northern detection and hybridization conditions.

gene	probe	hybridization temperature (°C) / buffer
micA	Riboprobe generated from PCR product amplified with primers JVO-0236/ JVO-0266	42 / RAPIDhyb
rybB	oligo JVO-1205	45 / RAPIDhyb
ompA	dsDNA; PCR product amplified with primers JVO-0397 / JVO-0398	65 / RAPIDhyb
ompC	dsDNA, PCR product amplified with primers JVO-717 / JVO-719	65 / RAPIDhyb
ompD	Riboprobe generated from PCR product amplified with primers JVO-1057 / JVO-1058	70 / RAPIDhyb
ompF	dsDNA; PCR product amplified with primers JVO-0430 / JVO-1202	68 / RAPIDhyb
ompN	Riboprobe generated from PCR product amplified with primers JVO-1195 / JVO-1196	68 / RAPIDhyb
ompS	Riboprobe generated from PCR product amplified with primers JVO-1199 / JVO-1200	68 / RAPIDhyb
ompW	Riboprobe generated from PCR product amplified with primers JVO-1197 / JVO-1198	68 / RAPIDhyb
ompX	dsDNA; PCR product amplified with primers JVO-1187 / JVO-1188	68 / RAPIDhyb
fadL	Riboprobe generated from PCR product amplified with primers JVO-1203 / JVO-1204	68 / RAPIDhyb
sodB	dsDNA; PCR product amplified with JVO-0932 / JVO-0933	68 / RAPIDhyb
5S rRNA	oligo JVO-0322	45 / RotiQuick

Legend for Supplementary Figure S1: Northern blot showing that polymyxin B-induced downregulation of *ompD* mRNA in wild-type *Salmonella* is dependent on σ^E activation. RNA samples were taken from polymyxin B-treated wild-type and Δ*rpoE* cells in late exponential phase (OD_{600} of 1). Total RNA was prepared prior to (0 min) and after 5 and 10 min of polymyxin B addition.

Supplementary References

Tusher, V.G., R. Tibshirani, and G. Chu. 2001. Significance analysis of microarrays applied to the ionizing radiation response. *Proc Natl Acad Sci U S A* 98: 5116-21.

Figure S1

Papenfort *et al.*, 2006

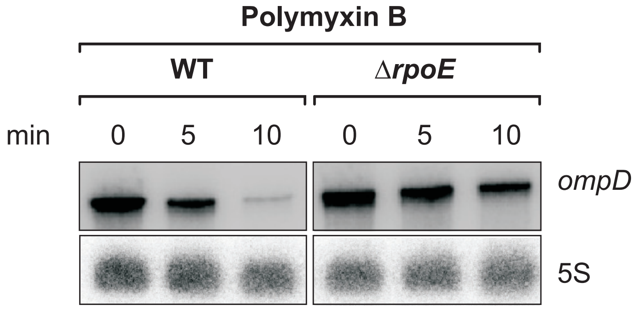

Supplement: Fig. S1 — An rpoE deletion abrogates the rapid downregulation of ompD mRNA upon polymyxin B treatment. [file mmi0062-1674-S1.pdf]
